# Supplementary material for: Hedgehog signaling via its ligand DHH acts as cell fate determinant during skeletal muscle regeneration
Source: Nat Commun. 2023 Jun 24;14:3766. doi: 10.1038/s41467-023-39506-1 (PMC10290686; doi:10.1038/s41467-023-39506-1)
Supplement: Supplementary file 1 — Supplementary Information [file 41467_2023_39506_MOESM1_ESM.pdf]

Hedgehog signaling via its ligand DHH acts as cell fate determinant during skeletal muscle regeneration

Alessandra M. Norris[1], Ambili Bai Appu[1], Connor D. Johnson[1], Lylybell Y. Zhou[1], David W. McKellar[2], Marie-Ange Renault[3], David Hammers[1], Benjamin D. Cosgrove[2] and Daniel Kopinke[1*].

[1]Department of Pharmacology and Therapeutics, Myology Institute, University of Florida, Gainesville, FL, USA.

[2]Meinig School of Biomedical Engineering, Cornell University, Ithaca, NY, USA

[3]Biology of Cardiovascular Diseases, INSERM, University of Bordeaux, Pessac, France

[*]Correspondence: dkopinke@ufl.edu

Supplemental Figures and Table

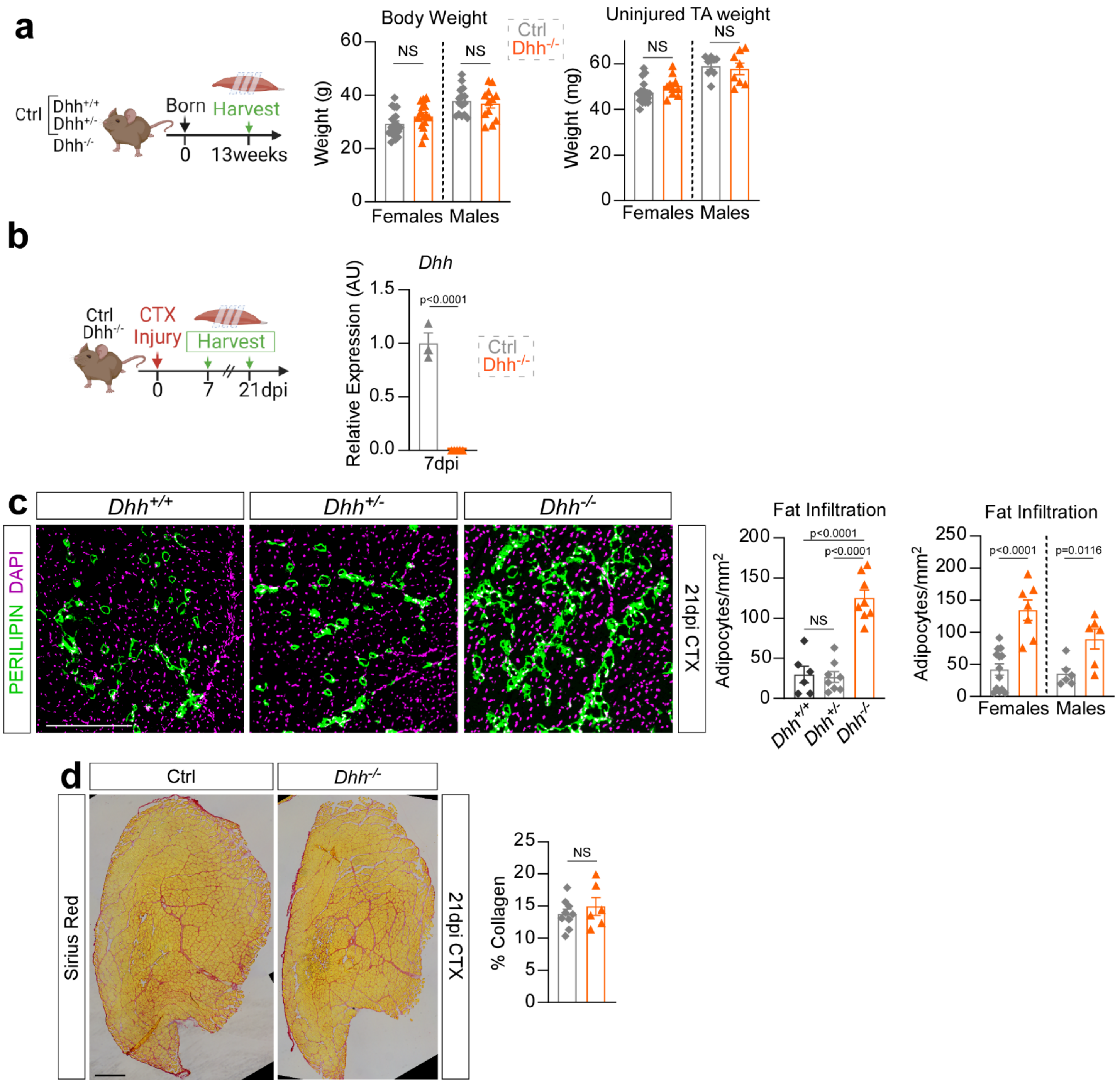

Supplemental Figure 1. Validation of the *Dhh*$^{-/-}$ mouse model.

a) Experimental outline. Body weight (g) of 13-week-old *Dhh*$^{-/-}$ (females, n=15 mice; males, n=12 mice) and ctrl mice (females, n=19 mice; males n=15 mice). Wet-weight of uninjured TAs (mg) of *Dhh*$^{-/-}$ (females, n=10 TAs; males, n=8 TAs) and ctrl mice (females, n=17 TAs; males n=9 TAs). **b)** Experimental outline. RT-qPCR of *Dhh* expression 7 days after CTX injury of *Dhh*$^{-/-}$ (n=5 TAs) and ctrl (n=3 TAs) mice. **c)** *Left*: Immunofluorescence of adipocytes (PERILIPIN^{+}, green) 21 days post CTX injury of *Dhh*$^{+/+}$, *Dhh*$^{+/-}$ and *Dhh*$^{-/-}$ mice. Nuclei were visualized with DAPI (purple). Scale bars: 250 µm. *Right:* Quantification of adipocytes per injured area (mm^2) 21 days post CTX injury in *Dhh*$^{+/+}$ (n=6 TAs), *Dhh*$^{+/-}$ (n=8 TAs) and *Dhh*$^{-/-}$ (n=8 TAs) mice. Adipocyte quantification of *Dhh*$^{-/-}$ and ctrl mice separated by females (*Dhh*$^{-/-}$ n=7 TAs; ctrl n=13 TAs) and males (*Dhh*$^{-/-}$ n=6 TAs; ctrl n=6 TAs). **d)** *Left:* Histological Sirius red staining 21 days post CTX injury in *Dhh*$^{-/-}$ and ctrl mice. Scale bars: 500 µm. *Right:* Quantification of percent of collagen (red) over total TA area 21 dpi in *Dhh*$^{-/-}$ (n=6 TAs) and ctrl mice (n=9 TAs). All data are represented as mean ± SEM. An unpaired two-tailed t test or a one-way ANOVA followed by a Dunnet's multiple comparison was used.

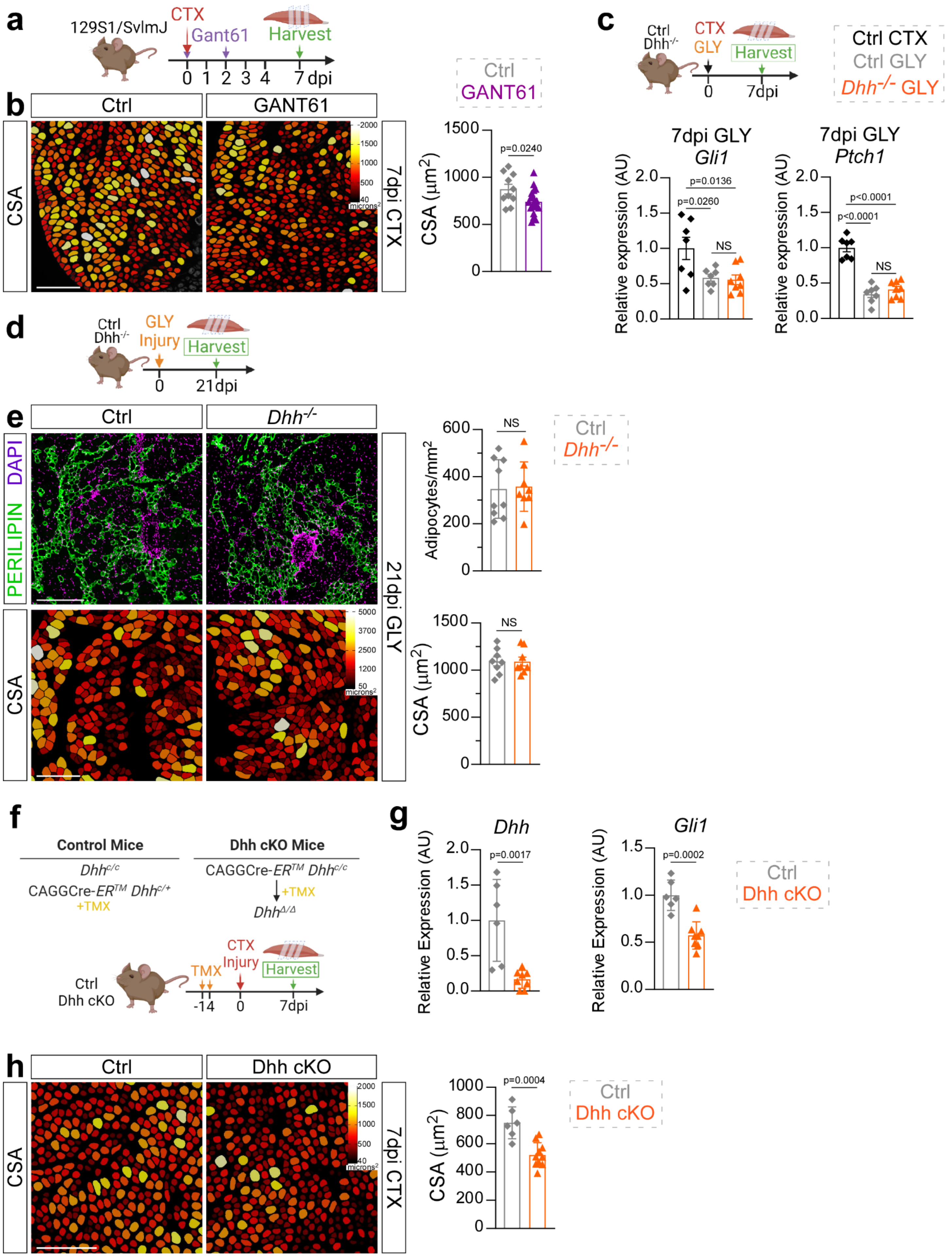

a
129S1/SvlmJ
CTX
Gant61
Harvest
0 1 2 3 4 7 dpi
b
Ctrl
GANT61
CSA
7dpi CTX
Ctrl
GANT61
CSA (μm2)
p=0.0240
c
Ctrl
Dhh-/-
CTX
GLY
Harvest
0 7dpi
Ctrl CTX
Ctrl GLY
Dhh-/- GLY
7dpi GLY Gli1
7dpi GLY Ptch1
Relative expression (AU)
p=0.0136
p=0.0260
NS
p<0.0001
p<0.0001
NS
d
Ctrl
Dhh-/-
GLY
Injury
Harvest
0 21dpi
e
Ctrl
Dhh-/-
PERILIPIN DAPI
CSA
21dpi GLY
Adipocytes/mm2
NS
Ctrl
Dhh-/-
CSA (μm2)
NS
f
Control Mice
Dhh cKO Mice
Dhhc/c
CAGGCre-ERTM Dhhc/+
+TMX
CAGGCre-ERTM Dhhc/c
+TMX
DhhΔ/Δ
Ctrl
Dhh cKO
TMX
CTX
Injury
Harvest
-14 0 7dpi
g
Dhh
Gli1
Relative Expression (AU)
p=0.0017
p=0.0002
Ctrl
Dhh cKO
h
Ctrl
Dhh cKO
CSA
7dpi CTX
CSA (μm2)
p=0.0004
Ctrl
Dhh cKO

Supplemental Figure 2. Validation of DHH loss of function data and Dhh cKO mouse model.
a) Experimental outline. **b)** *Left:* Myofibers color-coded according to size (μm^2) of Gant61- and vehicle treated mice 7 days post CTX injury. Scale bar: 250 µm. *Right*: Average CSA (μm^2) of vehicle (n=10 TAs) and Gant61 (n=19 TAs) treated mice 7 days post CTX. **c)** *Top*: Experimental outline. *Bottom*: RT-qPCR of expression of *Gli1* and *Ptch1* 7 days post CTX in ctrl (n=7 TAs); and 7 days post GLY injury in ctrl (n=7 TAs) and $Dhh^{-/-}$ (n=8 TAs) mice. **d)** Experimental outline. **e)** *Left*: Immunofluorescence of adipocytes (PERILIPIN$^+$, green) and nuclei visualized with DAPI (purple); color-coded myofibers according to CSA of $Dhh^{-/-}$ and ctrl mice 21 days post GLY. Scale bars: 250 µm. *Right*: Quantification of adipocytes per injured area (mm^2) 21 days post GLY in $Dhh^{-/-}$ (n=8 TAs) and ctrl (n=9 TAs) mice. Average CSA (μm^2) of $Dhh^{-/-}$ (n=8 TAs) and ctrl (n=8 TAs) mice 21 days after GLY injury. **f)** Experimental outline. **g)** RT-qPCR of expression of *Dhh* and *Gli1* 7 days post CTX in Dhh cKO (n=8 TAs) and ctrl (n=6 TAs) mice. **h)** *Left*: Color-coded myofibers according to CSA (μm^2) of Dhh cKO and ctrl 7 days post CTX injury. Scale bar: 250 µm. *Right*: Average CSA (μm^2) of Dhh cKO (n=10 TAs) and ctrl (n=6 TAs) mice 7 days post CTX injury. All data are represented as mean ± SEM. An unpaired two-tailed t test or a one-way ANOVA followed by a Dunnet's multiple comparison was used.

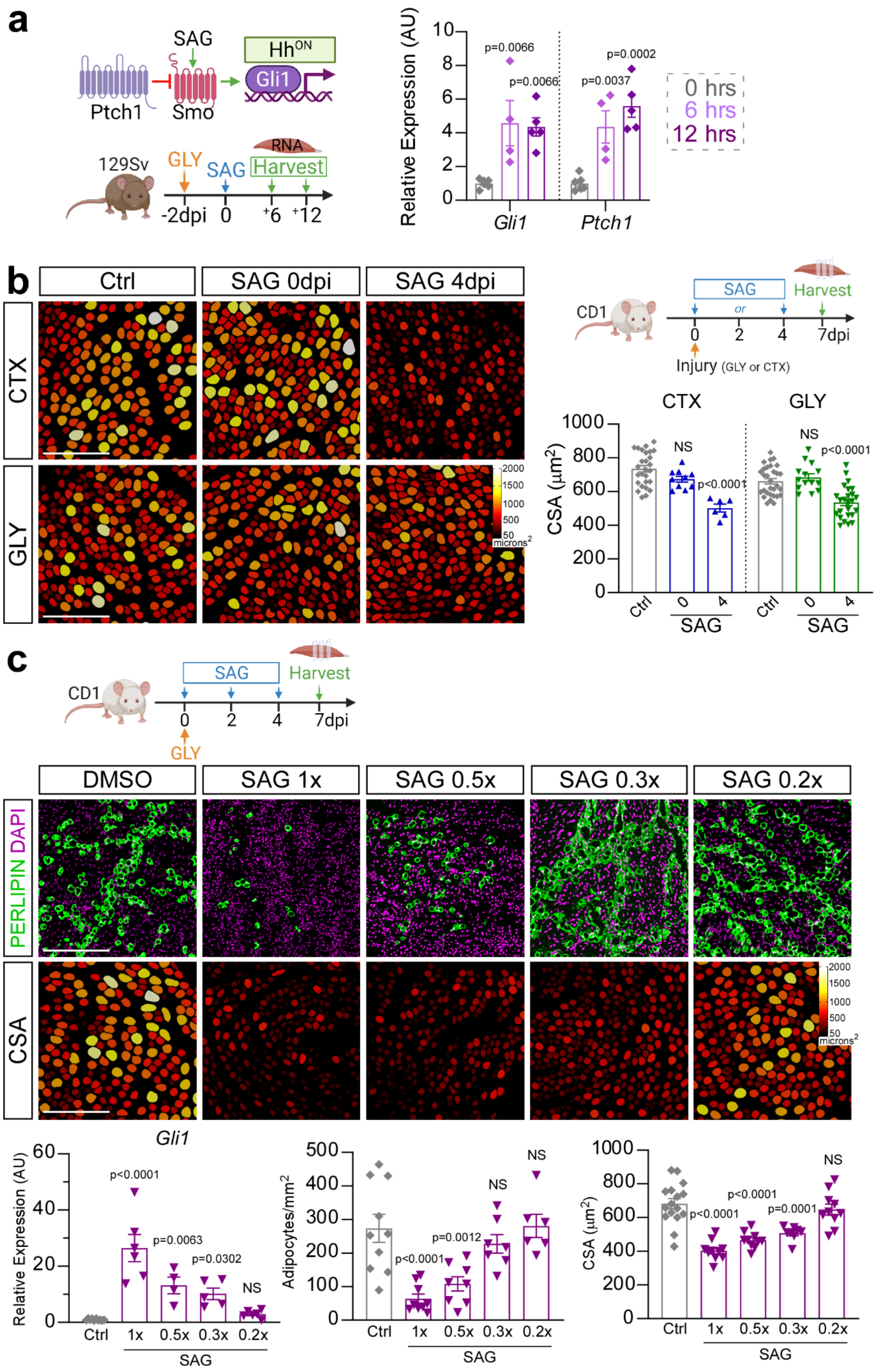

a
SAG
Hh^ON
Gli1
Ptch1
Smo
129Sv
GLY
SAG
RNA
Harvest
-2dpi
0
+6
+12
Relative Expression (AU)
p=0.0066
p=0.0066
p=0.0037
p=0.0002
Gli1
Ptch1
0 hrs
6 hrs
12 hrs
b
Ctrl
SAG 0dpi
SAG 4dpi
CTX
GLY
microns2
CD1
SAG
or
Harvest
7dpi
Injury (GLY or CTX)
CTX
GLY
CSA (µm2)
NS
p<0.0001
NS
p<0.0001
Ctrl
SAG
c
CD1
SAG
Harvest
7dpi
GLY
DMSO
SAG 1x
SAG 0.5x
SAG 0.3x
SAG 0.2x
PERLIPIN DAPI
CSA
microns2
Gli1
Relative Expression (AU)
p<0.0001
p=0.0063
p=0.0302
NS
Ctrl
1x
0.5x
0.3x
0.2x
SAG
Adipocytes/mm2
p<0.0001
p=0.0012
NS
NS
CSA (µm2)
p<0.0001
p<0.0001
p=0.0001
NS

Supplemental Figure 3. SAG influences adipogenesis and myogenesis in a dose- and time-dependent manner.

a) *Left:* Experimental outline. *Right*: After 2 days post GLY injury, RT-qPCR of *Gli1* and *Ptch1* at 0 hrs (n=6 TAs), 6 hrs (n=4 TAs) and 12 hrs (n=5 TAs) following SAG administration. **b)** *Left:* Myofibers color-coded according to size (μm^2) 7 days post CTX or GLY in ctrl and SAG- treated (at 0 dpi or 4 dpi) mice. Scale bar: 250 µm. *Right*: Average CSA (μm^2) 7 days post injury with vehicle control (CTX n=27 TAs & GLY n=30 TAs), SAG at 0 dpi (CTX: n=10 TAs & GLY: n=14 TAs) and SAG at 4 dpi (CTX: n=6 TAs & GLY: n=25 TAs). To note, these time points are part of the experiment described in the main Figure 3 and, thus, the same controls were used. **c)** SAG was administered at 0-, 2- and 4dpi at varying concentrations and TAs harvested at 7 days post GLY injury. Immunofluorescence of adipocytes ($PERILIPIN^+$, green) and nuclei visualized with DAPI (purple). Color-coded myofibers based on cross sectional area (CSA). Scale bars: 250 µm. *Bottom*: RT-qPCR of *Gli1* expression 7 days post GLY after treatment with DMSO control (n=10 TAs), SAG 1x (n=6 TAs), SAG 0.5x (n=4 TAs), SAG 0.3x (n=5 TAs) and SAG 0.2x (n=6 TAs). Quantification of adipocytes per injured area (mm^2) 7 days post GLY injury of DMSO control (n=10 TAs), SAG 1x (n=9 TAs), SAG 0.5x (n=8 TAs), SAG 0.3x (n=7 TAs), and SAG 0.2x (n=6 TAs). Average CSA (μm^2) 7 days after GLY injury of DMSO control (n=16 TAs), SAG 1x (n=10 TAs), SAG 0.5x (n=11 TAs), SAG 0.3x (n=8 TAs), and SAG 0.2x (n=10 TAs). All data are represented as mean ± SEM. One- way ANOVA followed by a Dunnet's multiple comparison was used.

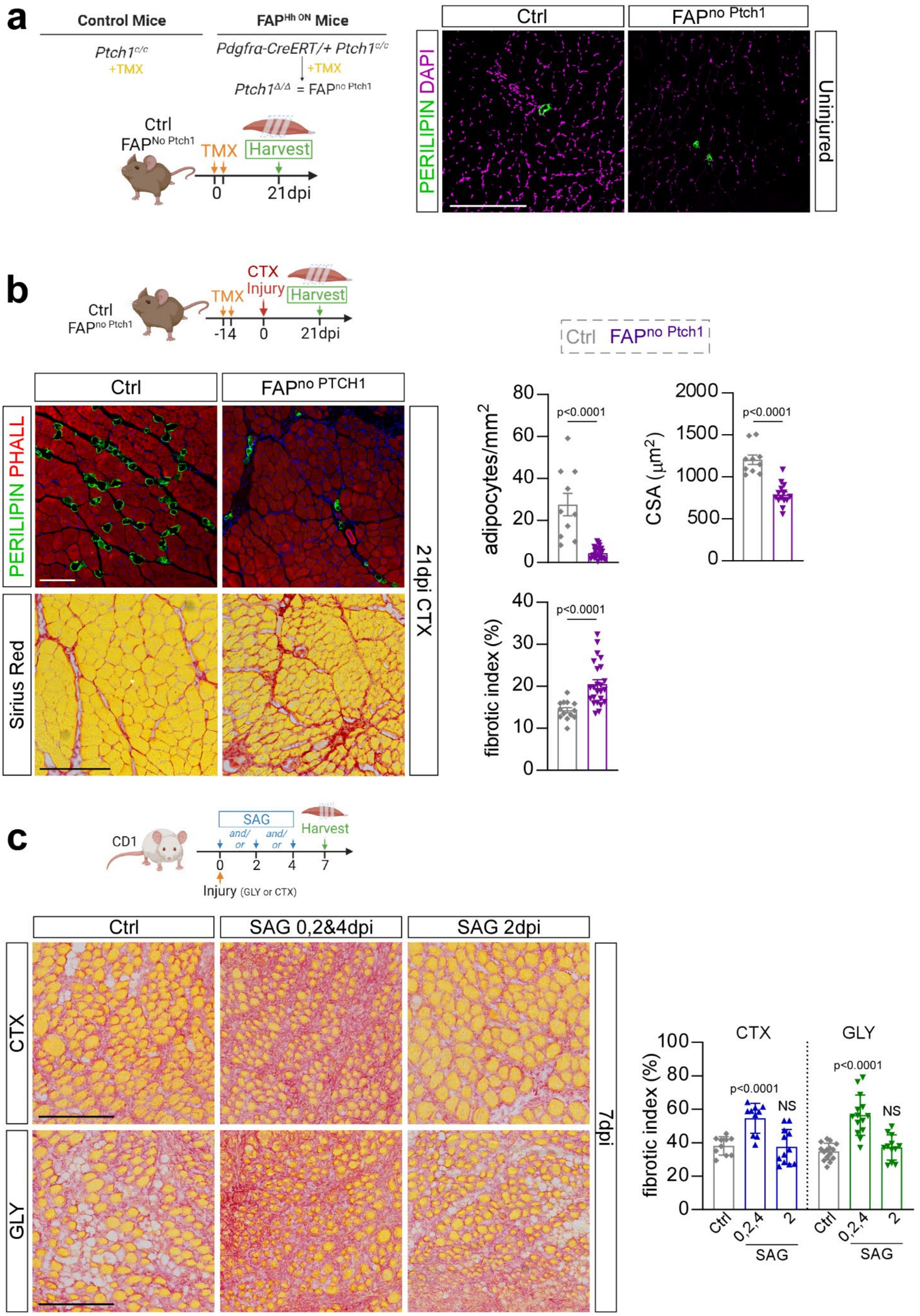

a
Control Mice
FAP^Hh ON Mice
Ptch1c/c
+TMX
Pdgfra-CreERT/+ Ptch1c/c
+TMX
Ptch1Δ/Δ = FAPno Ptch1
Ctrl
FAPNo Ptch1
TMX
Harvest
0
21dpi
Ctrl
FAPno Ptch1
PERILIPIN DAPI
Uninjured
b
Ctrl
FAPno Ptch1
TMX
CTX Injury
Harvest
-14
0
21dpi
Ctrl FAPno Ptch1
Ctrl
FAPno PTCH1
PERILIPIN PHALL
Sirius Red
21dpi CTX
adipocytes/mm2
p<0.0001
CSA (μm2)
p<0.0001
fibrotic index (%)
p<0.0001
c
CD1
SAG
and/or
and/or
Harvest
0
2
4
7
Injury (GLY or CTX)
Ctrl
SAG 0,2&4dpi
SAG 2dpi
CTX
GLY
7dpi
fibrotic index (%)
CTX
GLY
p<0.0001
NS
p<0.0001
NS
Ctrl
0,2,4
2
SAG
Ctrl
0,2,4
2
SAG

Supplemental Figure 4. Genetic and pharmacological Hh activation prevents IMAT formation but causes fibrosis and impairs myogenesis.

a) *Left*: Experimental outline. *Right:* Immunofluorescence of PERILIPIN$^+$ adipocytes (green) of uninjured FAP$^{no\ Ptch1}$ and control mice (n=5 for each). Scale bars: 250 µm. **b)** Immunofluorescence of PERILIPIN$^+$ adipocytes (green) and PHALLOIDIN+ myofibers (scale bar: 100 µm) and histological Sirius red staining (scale bar: 250 µm) 21 days post CTX injury in FAP$^{no\ Ptch1}$ and control mice. *Right*: Average CSA (µm^2) 21 days post CTX of ctrl (n=10 TAs) and FAP$^{no\ Ptch1}$ (n=14 TAs) mice. Quantification of adipocytes per injured area (mm^2) 21 days post CTX injury of ctrl (n=10 TAs) and FAP$^{no\ Ptch1}$ (n=22 TAs) mice. Quantification of percent of collagen (red) of total TA area 21 dpi CTX in FAP$^{no\ Ptch1}$ (n=19 TAs) and ctrl (n=24 TAs). **c)** *Left:* Sirius red staining 7 days after CTX *(top)* or GLY *(bottom)* injury after vehicle control, SAG at 0-, 2- and 4 dpi, or SAG at 2 dpi. Scale bars: 250 µm. *Right:* Quantification of percent of Sirius red-positive collagen (red) over total TA area 7 days after injury from mice treated with vehicle control (CTX: n=10 TAs & GLY: n=15 TAs), SAG at 0-, 2- and 4 dpi (CTX: n=10 TAs & GLY: n=14 TAs) or SAG at 2 dpi (CTX & GLY: n=12 TAs). **c)** All data are represented as mean ± SEM. An unpaired two-tailed t test or a one-way ANOVA followed by a Dunnet's multiple comparison was used.

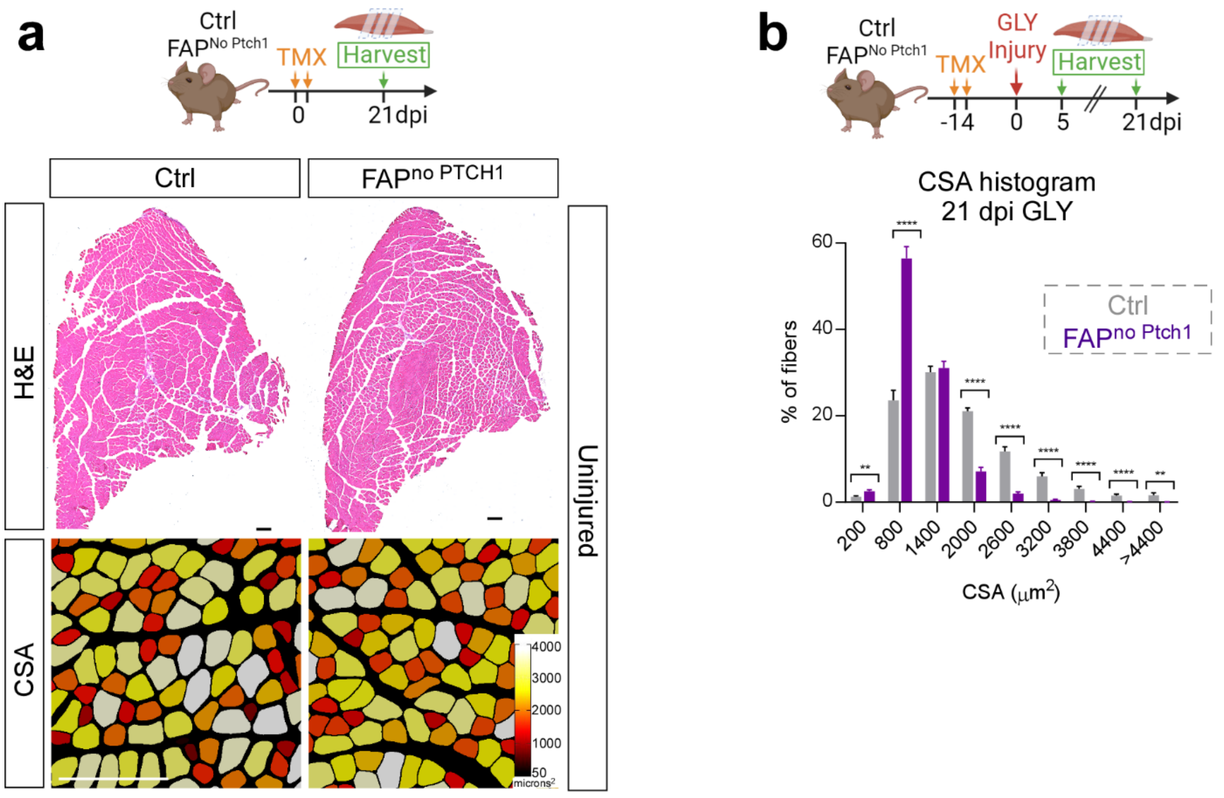

Supplemental Figure 5. Ectopic Hh activation does not impact muscle homeostasis.
a) Experimental outline. Color-coded myofibers based on cross sectional area (CSA) of uninjured FAP$^{no\ Ptch1}$ and control mice. Scale bar: 250 µm. H&E staining of uninjured FAP$^{no\ Ptch1}$ and control mice. Scale bar: 500 µm. **b)** *Top*: Experimental outline. *Middle*: Fiber number distribution, displayed as percent of total fibers, based on their CSA (µm^2) in FAP$^{no\ Ptch1}$ (n=18) and ctrl mice (n=14) 21 days post GLY injury. Source data are provided as a Source Data file. All data are represented as mean ± SEM. A two-way ANOVA followed by Tukey's multiple comparison test was used. A p value less than 0.05 was considered statistically significant where: ** p≤ 0.01, *** p≤ 0.001 and **** p≤ 0.0001. Source data are provided as a Source Data file.

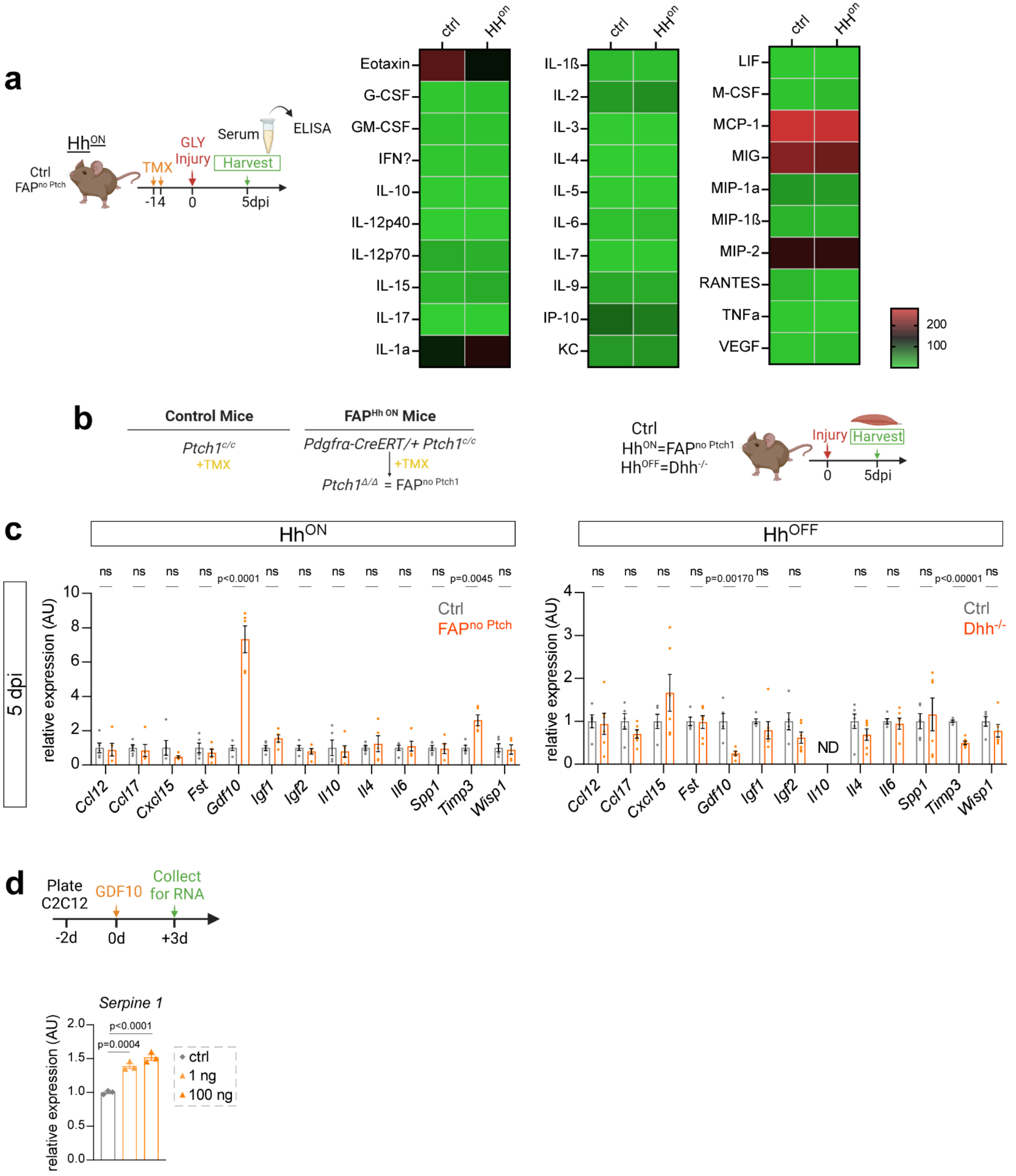
a
Hh^ON
Ctrl
FAP no Ptch
TMX
GLY
Injury
Serum
ELISA
Harvest
-14
0
5dpi
ctrl
HH on
Eotaxin
G-CSF
GM-CSF
IFN?
IL-10
IL-12p40
IL-12p70
IL-15
IL-17
IL-1a
IL-1ß
IL-2
IL-3
IL-4
IL-5
IL-6
IL-7
IL-9
IP-10
KC
LIF
M-CSF
MCP-1
MIG
MIP-1a
MIP-1ß
MIP-2
RANTES
TNFa
VEGF
200
100
b
Control Mice
FAP Hh ON Mice
Ptch1 c/c
+TMX
Pdgfra-CreERT/+ Ptch1 c/c
+TMX
Ptch1 Δ/Δ = FAP no Ptch1
Ctrl
Hh ON=FAP no Ptch1
Hh OFF=Dhh -/-
Injury
Harvest
0
5dpi
c
Hh ON
Hh OFF
5 dpi
relative expression (AU)
ns
p<0.0001
p=0.0045
p=0.00170
p<0.00001
Ctrl
FAP no Ptch
Dhh -/-
ND
Ccl12
Ccl17
Cxcl15
Fst
Gdf10
Igf1
Igf2
Il10
Il4
Il6
Spp1
Timp3
Wisp1
d
Plate C2C12
GDF10
Collect for RNA
-2d
0d
+3d
Serpine 1
relative expression (AU)
p<0.0001
p=0.0004
ctrl
1 ng
100 ng

Supplemental Figure 6. Screen for FAP-specific and Hh-induced factors that control adipogenesis and myogenesis.

a) Enzyme-linked immunosorbent assay (ELISA) of whole muscle protein lysates from FAP$^{no\ Ptch1}$ and ctrl mice 5 days post GLY injury. **b)** Experimental outline. **c)** RT-qPCR of whole muscle RNA from FAP$^{no\ Ptch1}$ (n=5 TAs) and ctrls (n=5 TAs); and *Dhh*$^{-/-}$ (n=6 TAs) and ctrls (n=4-6) 5 days post injury for the known FAP targets: *Ccl2, Ccl7, Cxcl5, Fst, Gdf10, Igf1, Igf2, Il10, Il4, Spp1, Timp3* and *Wisp1*. **d)** RT-qPCR of *Serpine 1* from RNA isolated from C2C12 cells (n= 3 replicates per experimental group) 3 days after induction in ctrl versus rGDF10-treated cells (1 ng & 100 ng). All data are represented as mean ± SEM. A one-way ANOVA followed by a Dunnet's multiple comparison was used.

Supplementary Table

Supplementary Table 1: List of primers used for RT-qPCR

Gene	Forward	Reverse
Hprt	CATAACCTGGTTCATCATCGC	TCCTCCTCAGACCGCTTTT
Pde12	ACCTTTTGGGTGCCAGTAGA	CCAGAGGTCATCTGTCCTTCA
Dhh	CGATGGCTAGAGCGTTCAC	GTACCCAACTACAACCCCGA
Timp3	TAGACCAGAGTGCCAAAGGG	CCAGGATGCCTTCTGCAAC
Gli1	GGTGCTGCCTATAGCCAGTGTCCTC	GTGCCAATCCGGTGGAGTCAGACCC
Ptch1	AATTCTCGACTCACTCGTCCA	CTCCTCATATTTGGGGCCTT
Gdf10	CCAAATCCTTTGACGCCTACT	GCTCTGACGATGCTCTGGAT
Acta2	GACAGAGGCACCACTGAACC	ACAGCACAGCCTGAATAGCC
Ccl12	TTAAAAACCTGGATCGGAACCAA	GCATTAGCTTCAGATTTACGGGT
Ccl17	GCTGCTTTCAGCATCCAAGTG	CCAGGGACACCGACTACTG
Cxcl15	GTTCCATCTCGCCATTCATGC	GCGGCTATGACTGAGGAAGG
Fst	GCCTCCTGCTGCTGCTACTC	TTATACAGGACCTGGCAGCG
Igf1	CTGAGCTGGTGGATGCTCTT	TCATCCACAATGCCTGTCTG
Igf2	GTGCTGCATCGCTGCTTAC	ACGTCCCTCTCGGACTTGG
Il10	AGCATTTGAATTCCCTGGGT	TTTTCACAGGGGAGAAATCG
Il4	GGTCTCAACCCCCAGCTAGT	GCCGATGATCTCTCTCAAGTGAT
Il6	TAGTCCTTCCTACCCCAATTTCC	TTGGTCCTTAGCCACTCCTTC
Spp1	AGCAAGAAACTCTTCCAAGCAA	GTGAGATTCGTCAGATTCATCCG
Wisp1	GTGTGATGATGACGCAAGGA	ATGCAGTTCTCATACCGTTGC
Serpine 1	GTGAATGCCCTCTACTTCAGTG	GCTGCCATCAGACTTGTGGAA
