# Supplementary material for: Hedgehog signaling via its ligand DHH acts as cell fate determinant during skeletal muscle regeneration
Source: Nat Commun. 2023 Jun 24;14:3766. doi: 10.1038/s41467-023-39506-1 (PMC10290686; doi:10.1038/s41467-023-39506-1)
Supplement: Supplementary file 3 — Reporting summary [file 41467_2023_39506_MOESM3_ESM.pdf]

nature portfolio

Corresponding author(s): Daniel Kopinke

Last updated by author(s): May 31, 2023

Reporting Summary

Nature Portfolio wishes to improve the reproducibility of the work that we publish. This form provides structure for consistency and transparency in reporting. For further information on Nature Portfolio policies, see our Editorial Policies and the Editorial Policy Checklist.

Statistics

For all statistical analyses, confirm that the following items are present in the figure legend, table legend, main text, or Methods section.

n/a	Confirmed	
☐	☒	The exact sample size (*n*) for each experimental group/condition, given as a discrete number and unit of measurement
☐	☒	A statement on whether measurements were taken from distinct samples or whether the same sample was measured repeatedly
☐	☒	The statistical test(s) used AND whether they are one- or two-sided<br>*Only common tests should be described solely by name; describe more complex techniques in the Methods section.*
☐	☒	A description of all covariates tested
☐	☒	A description of any assumptions or corrections, such as tests of normality and adjustment for multiple comparisons
☐	☒	A full description of the statistical parameters including central tendency (e.g. means) or other basic estimates (e.g. regression coefficient) AND variation (e.g. standard deviation) or associated estimates of uncertainty (e.g. confidence intervals)
☐	☒	For null hypothesis testing, the test statistic (e.g. *F*, *t*, *r*) with confidence intervals, effect sizes, degrees of freedom and *P* value noted<br>*Give P values as exact values whenever suitable.*
☒	☐	For Bayesian analysis, information on the choice of priors and Markov chain Monte Carlo settings
☒	☐	For hierarchical and complex designs, identification of the appropriate level for tests and full reporting of outcomes
☒	☐	Estimates of effect sizes (e.g. Cohen's *d*, Pearson's *r*), indicating how they were calculated

Our web collection on statistics for biologists contains articles on many of the points above.

Software and code

Policy information about availability of computer code

Data collection	Publicly available single-cell RNA sequencing data were downloaded and prepared as in McKellar et al, 2021 (DOI: 10.1038/s42003-021-02810-x). Briefly, 111 single-cell and single-nucleus RNA sequencing datasets were downloaded, aligned to the mm10 genome using cellranger (v3.1.0), preprocessed with Seurat (v3.2.1), and integrated using Harmony (v1.0).
Data analysis	All code used to prepare and analyze these data is available on github (https://github.com/mckellardw/scMuscle) and fully preprocessed data are available for download on Dryad (doi:10.5061/dryad.t4b8gtj34).

For manuscripts utilizing custom algorithms or software that are central to the research but not yet described in published literature, software must be made available to editors and reviewers. We strongly encourage code deposition in a community repository (e.g. GitHub). See the Nature Portfolio guidelines for submitting code & software for further information.

Data

Policy information about availability of data

All manuscripts must include a data availability statement. This statement should provide the following information, where applicable:

- Accession codes, unique identifiers, or web links for publicly available datasets
- A description of any restrictions on data availability
- For clinical datasets or third party data, please ensure that the statement adheres to our policy

Source data are provided with this paper. The code used to derive all scRNAseq data is available on github (https://github.com/mckellardw/scMuscle) and fully

preprocessed data are available on Dryad (doi:10.5061/dryad.t4b8gtj34). All other correspondence and material requests should be addressed to dkopinke@ufl.edu.

Human research participants

Policy information about studies involving human research participants and Sex and Gender in Research.

Reporting on sex and gender	n/a
Population characteristics	n/a
Recruitment	n/a
Ethics oversight	n/a

Note that full information on the approval of the study protocol must also be provided in the manuscript.

Field-specific reporting

Please select the one below that is the best fit for your research. If you are not sure, read the appropriate sections before making your selection.

☒ Life sciences ☐ Behavioural & social sciences ☐ Ecological, evolutionary & environmental sciences

For a reference copy of the document with all sections, see nature.com/documents/nr-reporting-summary-flat.pdf

Life sciences study design

All studies must disclose on these points even when the disclosure is negative.

Sample size	No sample-size quantification was performed. Sample numbers were used according to accepted standards in the field.
Data exclusions	No data were excluded.
Replication	All experiments presented were repeated at least once and as indicated in the manuscript.
Randomization	As genetically modified mice were used, samples were organized based on genotype.
Blinding	Investigators were blinded during data analysis.

Reporting for specific materials, systems and methods

We require information from authors about some types of materials, experimental systems and methods used in many studies. Here, indicate whether each material, system or method listed is relevant to your study. If you are not sure if a list item applies to your research, read the appropriate section before selecting a response.

Materials & experimental systems

n/a	Involved in the study	
☐	☒	Antibodies
☐	☒	Eukaryotic cell lines
☒	☐	Palaeontology and archaeology
☐	☒	Animals and other organisms
☒	☐	Clinical data
☒	☐	Dual use research of concern

Methods

n/a	Involved in the study	
☒	☐	ChIP-seq
☒	☐	Flow cytometry
☒	☐	MRI-based neuroimaging

Antibodies

Antibodies used	All antibodies, and their detailed information, used in this study can be found in the material & methods section. Briefly, primary antibodies used were rabbit anti-Perilipin (1:1000; Cell Signaling, 9349S), rabbit anti-Laminin (1:1000; Sigma-Aldrich, L9393), rabbit anti-MyoG (1:250; Proteintech Group 14688-1-AP), rabbit anti-cleaved Caspse 3 (1:500, Millipore Sigma AB3623), anti-PAX7 (1:15; DSHB, AB 428528, supernatant), rat anti-BrdU (1:1000; Abcam AB6326), rabbit anti-MYH3 (1:250, Proteintech 22287-1-AP) and rat anti-MyoD (1:250; Invitrogen, MA1-41017) and goat anti-PDGFRα (1:250, R&D Systems #AF1062). Secondary antibodies used were

	Alexa Fluor-conjugated secondary antibodies from Life Technologies (1:1000) in combination with the directly conjugated dyes Phalloidin-Alexa 568 and 647 (1:200, Molecular Probes # A12380 & A22287).
Validation	We only used commercially available antibodies that were validated by manufacturer.

Eukaryotic cell lines

Policy information about cell lines and Sex and Gender in Research

Cell line source(s)	C2C12 myoblast cell line was derived from ATCC.
Authentication	Cells were tested to undergo successful myogenesis, which they did at high efficancy.
Mycoplasma contamination	Cells were regularly tested.
Commonly misidentified lines (See ICLAC register)	n/a

Animals and other research organisms

Policy information about studies involving animals; ARRIVE guidelines recommended for reporting animal research, and Sex and Gender in Research

Laboratory animals	B6;129S-Dhhtm1Amc/J (Dhh-/- mice, Jax# 002784), 129S1/SvlmJ , Cd1, CAGGCre-ERTM (Jax# 004682), PdgfraCreERT2 allele (Jax# 032770), Ptch1tm1Bjw (Jax: 030494), Dhhlox/lox (Caradu, et. al, 2018). All mice were used between 2-4 months of age. Mice were housed in standard ventilated cages at controlled temperature (22–23°C), 40-50% humidity, 12-h light/dark cycle, and ad libitum access to food and water.
Wild animals	n/a
Reporting on sex	We carefully explored any potential sex differences and found none, which we reported in this manuscript.
Field-collected samples	n/a
Ethics oversight	All animal work was approved by the Institutional Animal Care and Use Committee (IACUC) of the University of Florida.

Note that full information on the approval of the study protocol must also be provided in the manuscript.
